# Experimental study on seepage characteristics of microfracture with different aperture

**DOI:** 10.1038/s41598-020-62350-y

**Published:** 2020-03-25

**Authors:** Shuai Zhang, Weiguo Qiao, Yue Wu, Zhenwang Fan, Lei Zhang

**Affiliations:** 1 Shandong Provincial Key Laboratory of Civil Engineering Disaster Prevention and Mitigation, Shandong University of Science and Technology, Qingdao, 266590 China; 2 College of Civil Engineering and Architecture, Shandong University of Science and Technology, Qingdao, 266590 China

**Keywords:** Hydrology, Solid Earth sciences

## Abstract

Exploring the flow mechanism of fluid in rock mass is important in solving the water inrush problems during tunnel excavation. However, it is difficult to obtain an undisturbed rock mass from the actual site conditions and study the flow mechanism of fluid through a fracture network composed of multiple single fractures. Therefore, a solution to simulate rock seepage using rock-like samples with single microfracture was presented in this paper. Water flow through microfracture was tested and the deformation of microfracture was recorded by quasi-distributed fiber Bragg grating (FBG) technology. Experimental data showed that Forchheimer’s law and Izbash’s law could well describe the nonlinear relationship between flow velocity and hydraulic gradient. The coefficient *b* in Forchheimer’s equation decreased with the increase of microfracture aperture. A critical value of E = 0.8 was proposed to classify the nonlinear flow regime: weak turbulence (E < 0.8) and fully developed turbulence (E > 0.8).

## Introduction

Investigation on seepage characteristics of rock mass plays an essential role in solving the problem of water inrush during the construction and development of underground engineering, for example geothermal excavation, construction of dam foundations, oil or shale gas exploitation, storage of nuclear waste, contaminant pollution control, groundwater utilization and grouting engineering^1–3^. When the fluid contacts with the rock mass, the geometry of rock fracture, the physical and mechanical properties of rock mass, the boundary stress and fluid pressure affect the seepage characteristics^4,5^. Therefore, it is of great significance to study the fluid-structure interaction of single joint.

In the past decade, a lot of research has been done by scholars to study the fluid-structure interaction of fractured rock mass as reported in the following. Cubic law is the most popularity single joint seepage model. It simplifies the rough joint as two ideal smooth parallel plates and reveals the seepage characteristics of single joint^6^. In the cubic law, the permeability is only related to the fracture aperture. However, many researchers have found that flow capacity of the fractures in rock mass hinges on the number of fractures, the aperture of fractures, orientation of fractures and type of fracture infillings^7–9^. Moreover, it is quite complicated and difficult to clearly reveal the seepage characteristics in the fracture network. In the fracture network, the flow capacity depends mainly on the seepage characteristics of the most prominent single fracture^10^. For single fractured rock, the aperture of fracture is affected by normal stress^11^. Therefore, in order to reveal the seepage mechanism of rock mass, it is necessary to study the flow characteristics of a single fracture. Experimental results of stress and seepage coupling test conducted by Sun *et al*.^12^ on large-scale rock specimens under three-dimensional stress showed that the three-dimensional stress and water pressure have a significant effect on the fracture permeability coefficient. Specifically, the fracture permeability coefficient decreases with increasing three-dimensional stress, and increases with increasing water pressure. Zhang *et al*.^13^ designed a physical model of fractured rock mass with different fracture aperture for seepage test and found that the flow velocity decreases with increasing fracture aperture under stable hydraulic pressure difference. Zhang *et al*.^14^ investigated the seepage characteristics of single fracture and established a radiation flow model. However, it is difficult to obtain undisturbed rock mass only containing a single fracture from the deep locations.

In this context, a lot of efforts have been made to find suitable materials to simulate intack rocks. Liu *et al*.^15^ used gypsum and sand as aggregate, silicone oil and Vaseline as binder, developing a fluid-structure interaction similar material. Zhao *et al*.^16^ developed a solid-gas coupling simulation material with sand as aggregate, oil and paraffin as binder. Experimental result of melt segregation and migration tests in rock conducted by Barraud *et al*.^17^ showed that paraffin is a good similar material to simulate the rock mass. Singh *et al*.^10^ found that paraffin is a suitable analog material to simulate the seepage process of single fractured rock mass in the laboratory environment.

At present, quantitative research on the flow characteristics of fractured rock mass mostly concentrates on obtaining the seepage discharge to study the flow behavior. However, few researchers focused on the deformation characteristics of rock fractures during the seepage process mainly due to the lack of suitable deformation sensors. The traditional metal sensor in bad geological environment has some defects, such as susceptible to moisture, rust failure, poor durability, poor contact, low survival rate and so on, which can’t be monitored automatically in real time. In recent years, distributed optical fiber sensing technology has been developed vigorously in basic research, equipment development and engineering application. In the monitoring of building structure, geotechnical engineering and geological engineering, the distributed optical fiber sensing technology has significant advantages, especially in the monitoring of rock and geological engineering, such as tunnel, foundation and dam^18,19^. Compared with the traditional monitoring technology, optical fiber is not only a sensing medium but also a transmission channel, which can be used for continuous measurement in space and more conducive to the analysis of the stress and deformation distribution of the structure. It also has the advantages of high sensitivity, anti-electromagnetic interference, good electrical insulation and excellent long-term corrosion resistance. Mohamad *et al*.^20^ used the optical fiber sensing technology to monitor the deformation of pile in the construction process, and introduced the installation method and data processing method of the sensor in detail. They found that the measured strain, temperature and stress are in good agreement with the traditional measurement method. Wang *et al*.^21^ explored the feasibility of optical fiber sensing technology for monitoring deformation stability and early failure alarm of slope, and found that the monitoring effect of fiber attached to anchor and frame beam is better than that of fiber embedded directly in soil due to the poor coordination of deformation between fiber and soil. Zhang^22^ embedded a horizontal network of optical fiber sensor on the surface of the soil above the tunnel, and monitored the strain field of the soil. The results show that this technology can accurately monitor the dynamic development process of soil strain field during the excavation process, and can give early warning of soil deformation and failure.

In the present work, fluid flow through samples with three fracture apertures were studied. With this idea, paraffin wax was selected as an analog material of sandstone, and its details are presented in this study. The FBG sensors were adopted to monitor the microfracture deformation in the fluid flow tests. In addition, the variations of normal deformation under different conditions were studied. Further, the nonlinear flow characteristics of samples with three different apertures were discussed. It is believed that this study provides a new technology and idea for fracture seepage simulation.

## Materials and Experiments

### Materials

In this research, the main objective is to simulate the microfracture seepage with different aperture under incremental change in normal and hydraulic pressures applying to samples. The paraffin wax was used as analog material of sandstone, which can ensure water flow mainly through the microfracture, but can’t through the matrix. Therefore, this method can help in better understanding of the basic principles of a single microfracture flow and the effects of various parameters (microfracture aperture, normal pressure, hydraulic pressure) on it. It should be noted that the method in creating certain mechanical aperture of the samples was inserting stainless-steel belt. The physical and mechanical parameters of paraffin wax are listed in Table 1.

**Table 1:** Physical and mechanical parameters of paraffin wax.

Compressive strength (MPa)	Tensile strength (MPa)	Modulus of elasticity (MPa)	Poisson’s ratio	Density (g/cm^3^)
1.7	0.5	227	0.36	0.89

### Sample preparation

**Figure 1:**
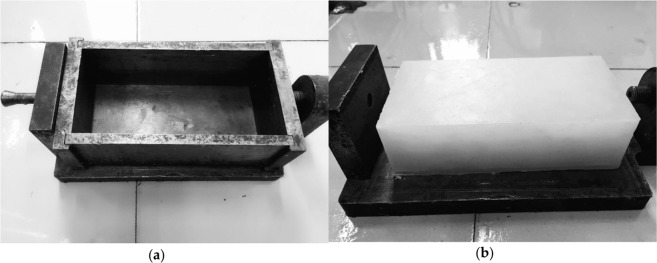
Sample preparation: (**a**) mold for sample preparation; (**b**) half of the sample.

In this experiment, paraffin wax samples were made to simulate the water flow through single microfracture, using the mold of 200 mm in length, 100 mm in width and 50 mm in height (Fig. 1a). After the molten paraffin wax was poured into the mold, it was placed in the laboratory to avoid external interference and ensure solidification and temperature uniformity. In this way, halves of the paraffin wax samples were prepared, as shown in Fig. 1b. The samples for seepage test were made up of two cuboid halves of the paraffin wax samples, and then stainless-steel belt (30 µm, 60 µm, 90 µm of thickness) was inserted in the middle of the two halves of paraffin wax samples to create microfracture with certain aperture.

### Experimental procedures

**Figure 2:**
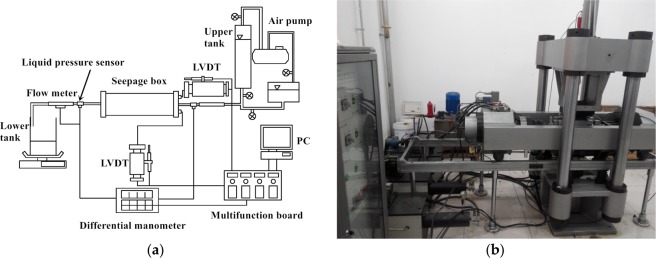
Photograph of JAW-600 fluid-structure interaction test system: (**a**) hydraulic test mechanism; (**b**) JAW-600 fluid-structure interaction test system.

The single microfracture seepage test of wax samples with the application of normal stress and hydraulic pressure is conducted by using a JAW-600 microcomputer-controlled test system. In this experiment, the test system contains the normal-force loading system, horizontal-force loading system, hydraulic pressure system, seepage box, and other components. The system diagram of the fundamental hardware configuration of this device is described in Fig. 2. The instrument can provide 30 MPa as the maximum normal pressure, and the hydraulic pressure is 3 MPa. The test of fluid flow through a microfracture at different combinations of hydraulic pressure and normal pressure as presented in Table 2.

**Table 2:** Test conditions of different load combinations.

Cases	Initial aperture (mm)	Hydraulic pressure h_p_ (MPa)	Normal pressure σ_3_ (MPa)
Case 1	0.03	0.8	0.2
Case 2	0.06	0.8	0.2
Case 3	0.09	0.8	0.2
Case 4	0.03	1.2	0.2
Case 5	0.03	1.6	0.2
Case 6	0.03	0.8	0.4
Case 7	0.03	0.8	0.6

**Figure 3:**
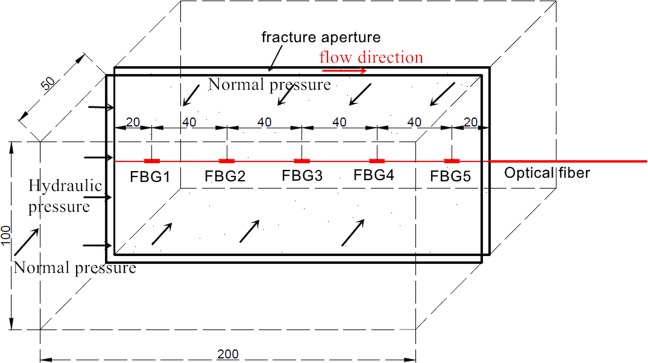
Photograph of FBG sensors installed in the sample (unit: mm).

**Figure 4:**
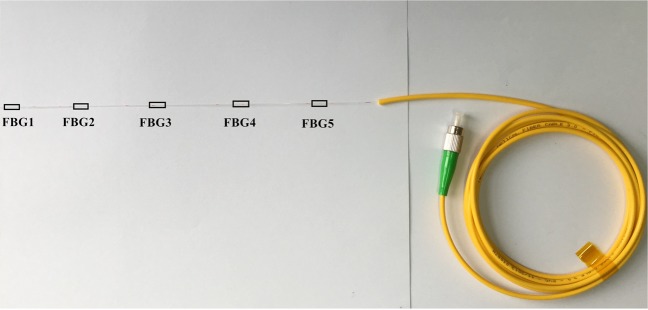
The structure diagram of the FBG sensors.

The seepage test procedures are described as follows:

(1) A groove with width and depth of 0.1 mm was firstly set on the microfracture surface by numerical control cutting technology. Then, 75% alcohol was used to wipe the groove. Afterwards, the FBG sensors were fixed in the groove by optical fiber adhesive, which the has the characteristics of high precision fixation, high heat resistance, high expansion and low water permeability. The FBG sensors have five measuring points to monitor the deformation and pressure of the fracture, as shown in Fig. 3. The typical measuring parameters of FBG sensors are listed in Table 3. Arrangement of FBG sensors on the microfracture surface is presented in Fig. 4. The ambient temperatures of all measurements were 20 °C and the temperature change less than 0.5 °C. Accordingly, the effet of temperature compensation in strain measurement can be ignored.

**Table 3:** Typical measuring parameter of the FBG sensors.

Wavelength range (nm)	Wavelength repeatability (nm)	Scan frequency (Hz)	Strain range (με)
1510–1590	0.001	1000	−3000 to 3000

**Figure 5:**
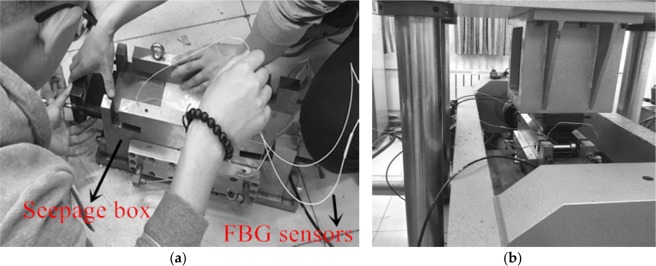
Test procedures: (**a**) Put sample into the seepage box; (**b**) installed the seepage box on the test platform.

(2) Put the sample into the seepage box and installed the seepage box on the JAW-600 test platform, as shown in Fig. 5. Afterwards, the normal force was applied to the seepage box, then hydraulic pressure was applied by hydraulic pressure system. Finally, collecting the test data by FBG sensors. An electronic balance with an accuracy of 0.01 g was placed at the outlet to measure the seepage discharge. During the test, the seepage discharge and the normal deformation of five measuring points were recorded every 3 s. The discharge was collected in an airtight container, which was placed on the high-precision electronic balance.

## Results and Discussion

### The variation of normal deformation of the microfracture under different initial aperture

**Figure 6:**
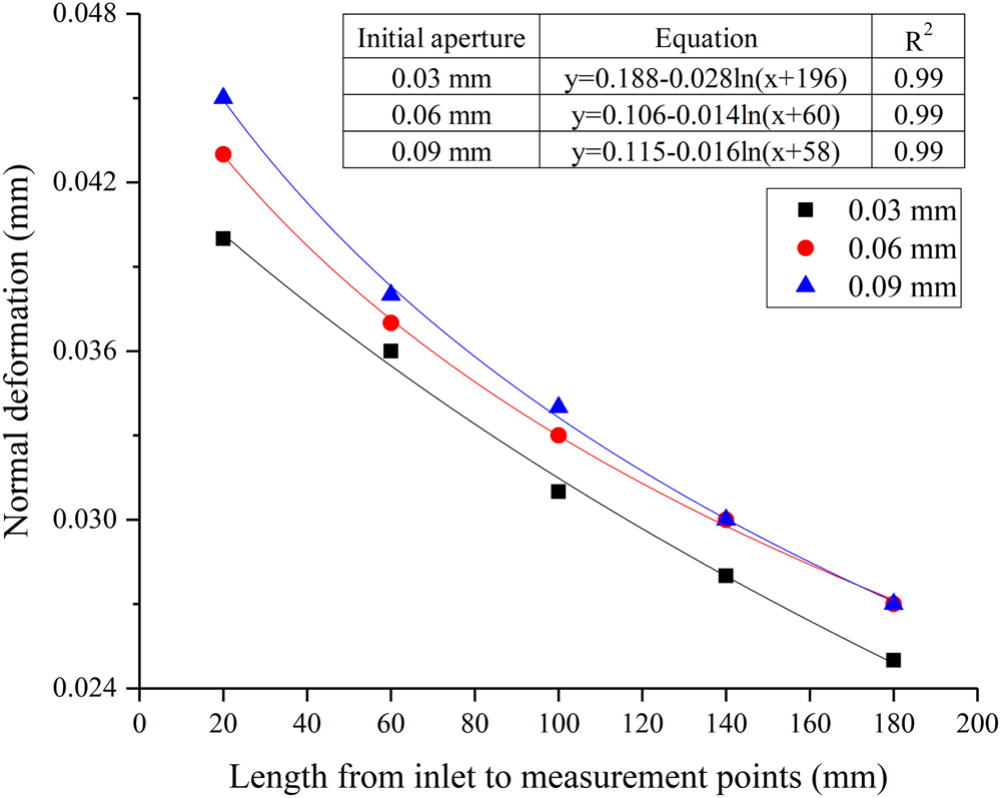
Variations of normal deformation for five measuring points with different initial aperture.

The application of external forces can result in the change of fracture aperture. The fracture of sample produces normal deformation due to the normal pressure and hydraulic pressure. The deformation of fracture with different aperture under same normal pressure and hydraulic pressure can be obtained according to the FBG sensors. The results in the form of the normal deformation versus the five measurement points under different initial aperture (hp = 0.8 MPa, σ_3_ = 0.2 MPa) are shown in Fig. 6.

From Fig. 6, it can be seen that there is a nonlinear relationship between normal deformation and seepage path. This relationship was in good agreement with logarithm function, and can be expressed by:

1 $${\rm{y}}={\rm{a}}-{\rm{b}}\ast \mathrm{ln}\,({\rm{x}}+{\rm{c}})$$

where a, b, c are the fitting parameters. It can be seen from Fig. 6 that the correlation coefficients are larger than 0.99. It can also be observed from the trends presented in Fig. 6 that the normal deformation has a negative correlation with the increase of seepage path. Specifically, the normal deformation decreases nonlinearly with the increase of seepage path. It may be due to the existence of resistance in the process of seepage, resulting in the gradual decrease of hydraulic pressure along the seepage path. However, the fracture aperture shows an increasing trend and the increment is equal to the normal deformation. This can be attributed to the fact that the loading of larger hydraulic pressure (compared with normal pressure), resulting in microfracture expansion and normal deformation increment. It can also be seen that there is little difference in normal deformation under the three cases. When the normal pressure and hydraulic pressure are same, the increments in the aperture of microfracture (0.03 mm, 0.06 mm and 0.09 mm) are 0.025–0.04 mm, 0.027–0.043 mm, and 0.027–0.045 mm, respectively. This indicates that microfracture initial aperture does not contributed to the normal deformation, which is mainly influenced by the difference between normal pressure and hydraulic pressure.

### The variation of normal deformation of the microfracture under different hydraulic pressures

**Figure 7:**
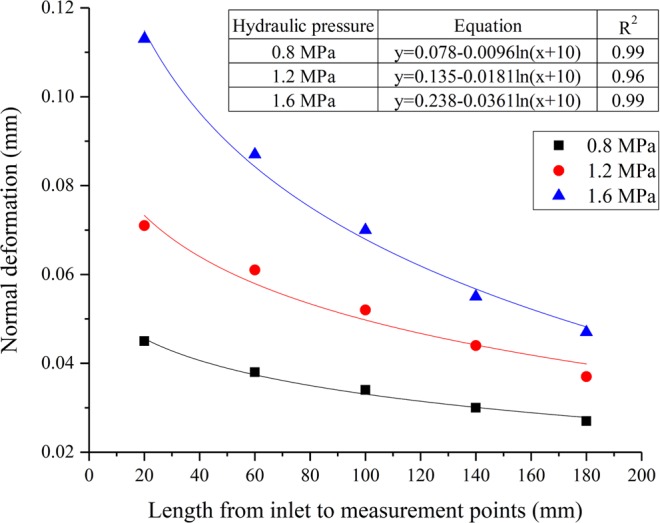
Variations of normal deformation for five measuring points with different hydraulic pressures.

The normal deformation of five measuring points corresponding to case 1, case 4 and case 5 was plotted in Fig. 7. It can be noticed from the figure that normal deformation of five measuring points increases non-linearly with the seepage path. When the hydraulic pressure values are 0.8 MPa, 1.2 MPa and 1.6 MPa, the ranges of normal deformation for five measuring points are 0.027–0.045 mm, 0.037–0.071 mm and 0.047–0.113 mm, respectively. It can be concluded that the hydraulic pressure is quite important to the normal deformation of microfracture. Further, the variation in normal deformation for five measuring points is not obvious when hydraulic pressure is less than 0.8 MPa.

### The variation of normal deformation of the microfracture under different normal pressures

**Figure 8:**
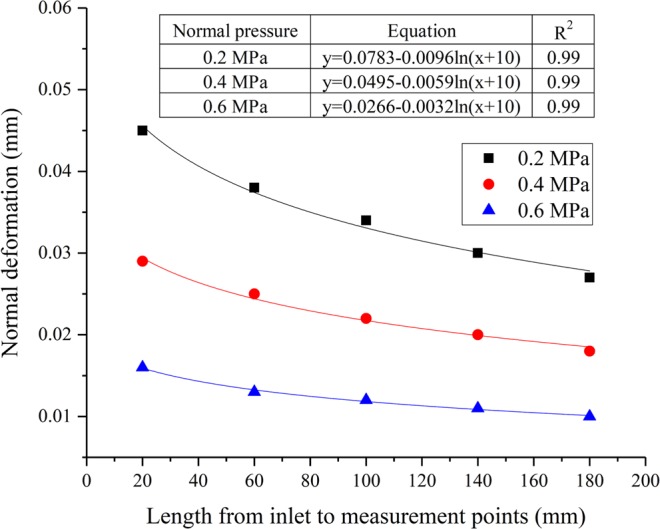
Variations of normal deformation for five measuring points with different normal pressures.

The variation in normal deformation of five measuring points corresponding to case 1, case 6 and case 7 has been plotted as shown in Fig. 8. It can be seen from Fig. 8 that normal deformation of five measuring points decreases non-linearly with seepage path and the expressions have been given. Moreover, the change in normal deformation tends to decrease with the normal force. When the normal pressure values are 0.2 MPa, 0.4 MPa and 0.6 MPa, the ranges of normal deformation for five measuring points are 0.027–0.045 mm, 0.018–0.029 mm and 0.01–0.016 mm, respectively.

### Seepage characteristics of microfracture with different aperture

**Figure 9:**
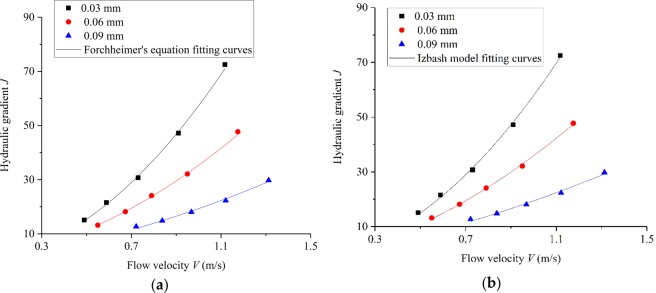
Relationship between *J* and *V* corresponding to different aperture: (**a**) Forchheimer’s law fitting curves; (**b**) Izbash’s law fitting curves.

The experimental data were analyzed and the relationship between hydraulic gradient *J* and average flow velocity *V* was investigated. The fracture aperture is the vertical distance between the two walls of the microfracture and calculated as the sum of normal deformation (obtained by the tests) and initial aperture. Thus, the true microfracture aperture was used to calculate the *V* in this paper, and it can be expressed by:

2 $${\rm{V}}=Q/(W\ast {e}_{{\rm{a}}})$$

where *Q* is the volumetric flow rate, *W* is the fracture width and *e*_a_ is the average aperture. This parameter *e*_a_ can be calculated by:

3 $${e}_{{\rm{a}}}=e+{d}_{{\rm{n}}}$$

4 $${d}_{{\rm{n}}}={\sum }_{(i=1)}^{5}(a-{\rm{b}}\ast \mathrm{ln}({\rm{x}}+{\rm{c}}))/5$$

where *e* is the initial aperture, *d*_n_ is the average normal deformation, *a* and *b* are the fitting parameters, *x*_*i*_ is the abscissa of the *i*th data point. Therefore, the *V* can be calculated by Eqs. (2), (3) and (4). The variation in *J* with *V* for fluid flow through microfracture under different mechanical aperture conditions has been plotted as depicted in Fig. 9. In this experiment, the average flow velocity values range from 0.49 to 1.31 m/s. It can be observed that the measured experimental data for samples with different aperture deviate from the linear relationship between *J* and *V*. This indicates that fluid flow through microfracture don’t obey cubic law and this observation is in good agreement with reports by Singh *et al*.^10^. However, Witherspoon *et al*.^23^ reported that cubic law was found to be valid whether the fracture surfaces were held open or were being closed under stress. This difference can be attributed to the result of inertial effect or fracture dilation, with the increase of flow velocity, the effect of inertial force becomes more and more significant, and the relationship between hydraulic gradient and velocity deviates from the linear relationship more and more^12^. For samples with different aperture, with the increase of the fracture aperture, the increase rate of the average velocity decreases gradually, as has been reported in the literature^24^.

To study seepage in microfracture with different aperture, the Forchheimer’s law is introduced to investigate the test results and describe the nonlinear flow behavior. Forchheimer’s equation is commonly used to generalize an empirical flow equation and can be expressed by:

5 $$\begin{array}{l}J=aV+b{V}^{2}\end{array}$$

where *a* and *b* are the coefficients reflecting the nonlinear effect. According to Forchheimer’s Eq. (5), the regression analyses were performed on the experimental data and the results obtained by fitting Eq. (5) are listed in Table 4.

**Table 4:** Fitting results for Forchheimer’s equation under different aperture.

Initial aperture/mm	*a* (s/m)	*b* (s^2^/m^2^)	*R*^2^
0.03	4.16	53.17	0.992
0.06	10.32	25.01	0.993
0.09	9.73	9.59	0.991

As can be seen from Table 4, the correlation coefficient *R*^2^ of the experimental data for each case exceeded 0.99. This means that Forchheimer’s model can be used to describe the nonlinear flow characteristics of microfractures in this study. Moreover, the coefficient *b* decreases with the increase in aperture, and *a* is smaller than *b* for each sample. This phenomenon is consistent with the results of Qian *et al*.^25^, who applied Forchheimer’s equation to analyze the experiment results of seepage test. The quadratic coefficient *b* decreases with the increase of microfracture aperture, whereas there is no obvious correlation between coefficient *a* and microfracture aperture. A similar experiment by Zoorabadi *et al*.^26^ reported that the coefficient *b* decreased with the increase of fracture aperture, which was consistent with the experimental results.

Izbash’s model is another common equation to explain non-Darcy phenomenon, which can be expressed by the following equation:

6 $$J=m{V}^{n}$$

where *m* and *n* (dimensionless) are the Izbash coefficients and the value of coefficient *n* is commonly between 1 and 2. For the case of *n* = 1, Eq. (6) can be simplified to the Darcy’s model and the flow behavior is equivalent to linear flow. For *n* ≠ 1, Izbash’s model provides a well description of nonlinear flow. According to Izbash’s Eq. (6), the regression analyses were performed on the experimental data and the regression parameters *m* and *n* are summarized in Table 5.

**Table 5:** Fitting *m* and *n* for Izbash’s equation under different aperture.

Initial aperture/mm	*λ*	*n*	*R*^2^
0.03	57.92	1.95	0.998
0.06	35.90	1.72	0.998
0.09	19.41	1.50	0.991

As can be seen from Table 5, the correlation coefficient *R*^2^ of each curve exceeded 0.99, thereby indicating that Izbash’s model can also provide a well description of nonlinear flow characteristics of fluid flow through microfracure. It can also be seen from Table 5 that the evolution law of the coefficients *m* and *n* is similar to the coefficient *b* in Forchheimer’s equation with the increase of microfracture aperture. Moreover, the coefficient *n* is larger than 1.5, thus indicating that inertial effect is more significant than viscous effect. Therefore, it can be concluded that the flow behavior in this experiment belongs to a fully turbulent flow. Similar observation has been reported by Zhang and Nemcik^27^, the Izbash’s model can well describe the nonlinear flow characteristics in rock fracture, and the exponential value of *n* is higher than 1.66, and it is consistent with the results of this study.

Reynolds number *Re* represents the ratio of inertial force to viscous force, which is defined as follows in fracture seepage:

7 $$Re=\rho Q/\mu W$$

where *ρ* is the density, *Q* is the volumetric flow rate, *μ* is dynamic viscosity of water and *W* is the fracture width. According to Eq. (7), the Reynolds number were calculated based on the experimental data and the results are summarized in Table 6.

**Table 6:** Values of Reynolds number *Re*.

*Re* (0.03 mm)	*Re* (0.06 mm)	*Re* (0.09 mm)
14.7	33	64.91
17.66	40.39	75.39
21.9	47.4	87.1
27.3	57	100.86
33.53	70.51	118.11

**Figure 10:**
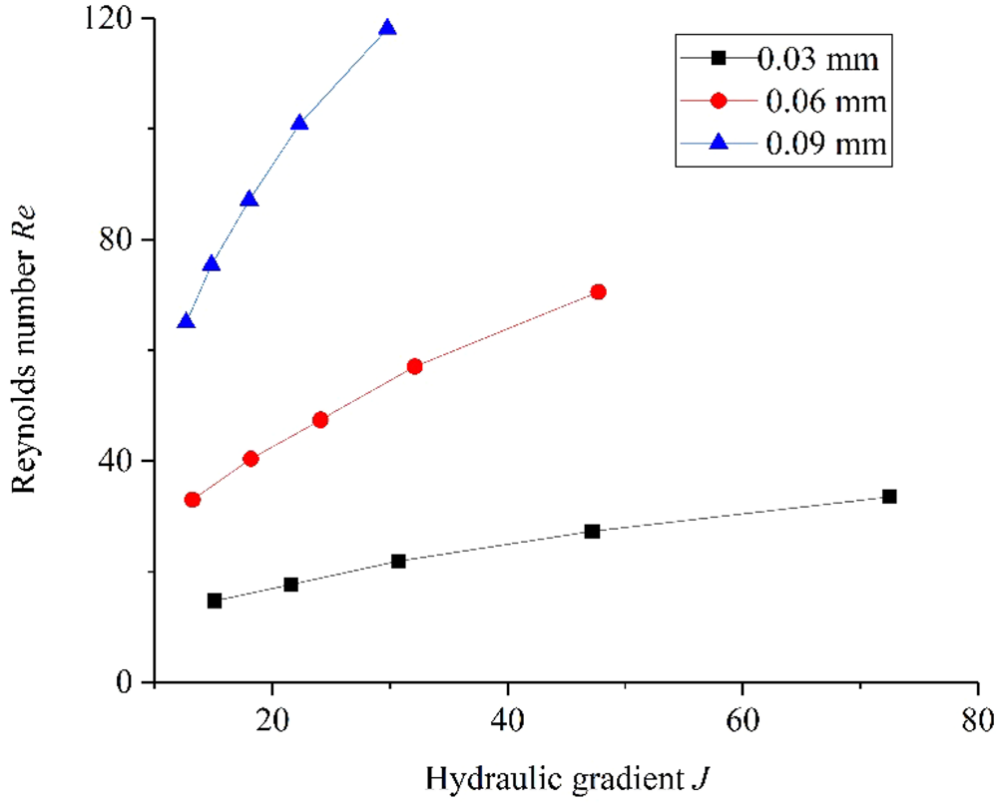
The curves in the form of *Re* and *J* under different aperture.

As can be seen from Table 6, for all cases, the Reynolds number values range from range 14.7 to 118.11. Hassanizadeh and Gary^28^ proposed a critical value of *Re* = 10 as the boundary between linear flow and turbulent. Table 6 shows that all of the Reynolds number values of seepage test exceed the critical value of *Re* = 10. Therefore, it can be concluded that the fluid flow through microfracture belongs to turbulence. To investigate the effect of microfracture aperture and hydraulic gradient on the *Re* more intuitively, the curves in the form of *Re* and *J* under three different apertures are plotted, as shown in Fig. 10.

It can be found from Fig. 10 that the Reynolds number is positive correlated with hydraulic gradient and there is a nonlinear relationship between Reynolds number and hydraulic gradient. It can be observed that with the increase of hydraulic gradient, the deviation of linear relationship between Reynolds number and hydraulic gradient becomes more remarkable. Specifically, the Reynolds number values increase with the increase of hydraulic gradient, while the increasing rate gradually reduces. Moreover, the slope of Reynolds number and hydraulic gradient increases with the increase of microfracture aperture.

**Figure 11:**
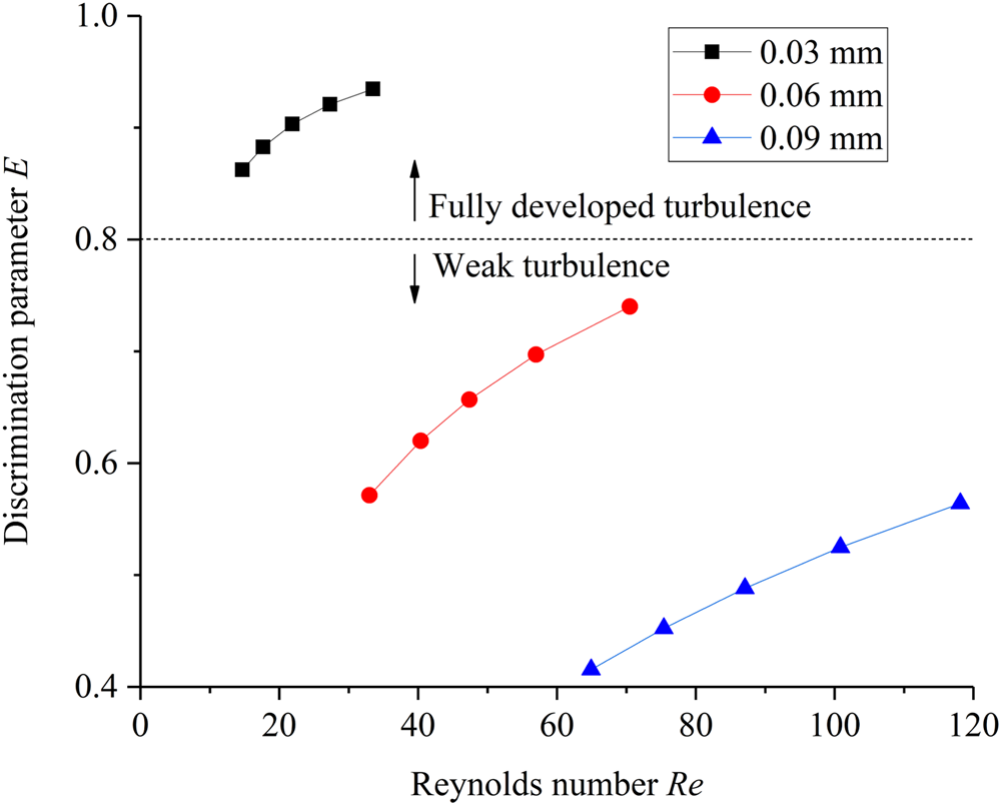
The curves in the form of E and Re under different aperture.

Even though the nonlinear flow characteristics of fluid flow through single microfracture have been investigated, while the threshold for the appearance of fully developed turbulence state needs further study. In order to quantitatively evaluate the nonlinear effect of fluid flow through single microfracture, a nonlinear factor E was proposed to evaluate the flow state, as expressed in Eq. (8):

8 $$E=l{V}^{2}/(rV+l{V}^{2})$$

where *rV* is the hydraulic head loss due to viscous force and *lV*^2^ is the hydraulic head loss due to inertia force. The discrimination parameter E is defined as the contribution of nonlinear term to total pressure gradient. In the case of E = 0, the inertia force can be ignored. This means that the fluid flow behavior of microfracture obeys Darcy’s law. In the case of E = 1, indicating that the fluid flow behavior of microfracture belongs to fully developed turbulence. The discrimination parameter E can be calculated according to Eq. (8), and the curves in the form of E and Re are shown in Fig. 11.

Zimmerman and Yeo^29^ proposed a critical value of *E* = 0.1 as the dividing point between linear flow and nonlinear flow for engineering purposes. It can be observed from Fig. 11 that all the discriminant factor *E* are higher than 0.4. This means that under the existing hydraulic gradient, the flow behavior of fluid in microfracture is obviously nonlinear. The results are in good agreement with reports by Qian *et al*.^25^, and stated that the discriminant factor was larger than 0.54 in their test. It can be attributed to the difference of fracture aperture (ranging from 0.5 to 2.0 mm) was larger than that in this study. In order to further classify the nonlinear flow state of microfracture, we determined a critical value of *E* = 0.8 to determine the state of fluid flow through a microfracture. In this case, the contribution of linear term to total pressure gradient is 20%. This research assumes that this region is a critical point, which indicates the location where viscous force can be disregarded and where the fully developed turbulence occurs. Therefore, the discriminant factor *E* = 0.8 can be used to divide the nonlinear flow state into two parts: weak turbulence (*E* < 0.8) and fully developed turbulence (*E* > 0.8). The critical value of *E* = 0.8 is smaller than obtained by of Xia *et al*.^29^, who reported that the flow sate from weak turbulence to genuine turbulence when the discriminant factor *E* = 0.9. This different finding can be attributed to the fact that the hydraulic gradient and fracture aperture in this study is much smaller than that of the latter. Therefore, this phenomenon can be considered as an extension of the results of Xia *et al*.^30^, establishing the relationship between hydraulic gradient and velocity in microfracture field. Although this result does not necessarily indicate whether the critical value of E = 0.8 can adequately prove linear flow or nonlinear flow in microfracture, while this critical value can be used as a reference for the study of fluid flow state in microfractures and this investigation proves the nonlinear flow distribution in microfracture.

## Conclusions

The main following conclusions can be drawn from the present study:

1. Nonlinear seepage characteristics were observed from the fluid flow through samples with microfracture. Both the Forchheimer’s law and Izbash’s law can be used to describe the non-Darcy flow characteristics caused by inertia effect in microfracture with different aperture.
2. The coefficient *b* in Forchheimer’s equation decreased with the increase of microfracture aperture. The evolution law of the coefficients *λ* and *n* in Izbash’s equation is similar to the coefficient b in Forchheimer’s equation with the increase of microfracture aperture.
3. A critical value of E = 0.8 was proposed to classify the nonlinear flow regime: weak turbulence (E < 0.8) and fully developed turbulence (E > 0.8).

## Data Availability

The datasets generated during and/or analysed during the current study are available from the corresponding author Weiguo Qiao on reasonable request.
